# Unique features and emerging in vitro models of human placental development

**DOI:** 10.1002/rmb2.12347

**Published:** 2020-09-12

**Authors:** Shun Shibata, Eri H. Kobayashi, Norio Kobayashi, Akira Oike, Hiroaki Okae, Takahiro Arima

**Affiliations:** ^1^ Department of Informative Genetics Tohoku University Graduate School of Medicine Sendai Japan

**Keywords:** DNA methylation, epigenetics, human placenta, organoid, trophoblast stem (TS) cells

## Abstract

**Background:**

The placenta is an essential organ for the normal development of mammalian fetuses. Most of our knowledge on the molecular mechanisms of placental development has come from the analyses of mice, especially histopathological examination of knockout mice. Choriocarcinoma and immortalized cell lines have also been used for basic research on the human placenta. However, these cells are quite different from normal trophoblast cells.

**Methods:**

In this review, we first provide an overview of mouse and human placental development with particular focus on the differences in the anatomy, transcription factor networks, and epigenetic characteristics between these species. Next, we discuss pregnancy complications associated with abnormal placentation. Finally, we introduce emerging in vitro models to study the human placenta, including human trophoblast stem (TS) cells, trophoblast and endometrium organoids, and artificial embryos.

**Main findings:**

The placental structure and development differ greatly between humans and mice. The recent establishment of human TS cells and trophoblast and endometrial organoids enhances our understanding of the mechanisms underlying human placental development.

**Conclusion:**

These in vitro models will greatly advance our understanding of human placental development and potentially contribute to the elucidation of the causes of infertility and other pregnancy complications.

## 1 INTRODUCTION

The placenta is a multifunctional organ that is essential for the survival and development of mammalian fetuses. The placenta contains both maternal and fetal cells and serves to provide nourishment and oxygen from the mother to the fetus. Besides, placental trophoblast cells are a major source of pregnancy‐related hormones and help protect the fetus from the immune system of the mother.^1^, ^2^, ^3^ It is interesting that the fetus, which is a semi‐allograft to the mother, is not attacked by the maternal immune cells in the uterus. The uterus is not an immunologically isolated organ, and many lymphocytes reside there. Thus, after implantation, embryos are exposed to maternal immune cells but do not trigger immune rejection. Abnormal placentation is associated with pregnancy complications such as infertility, miscarriage, and hypertensive disorders of pregnancy (HDP). These disorders are collectively called the “great obstetrical syndromes”.^4^ Although some pregnancy complications such as fetal growth restriction and HDP occur during the 2^nd^ and 3^rd^ trimester in humans, attention should be focused on the early stages of intrauterine development to understand the underlying mechanisms of these diseases. There is no doubt that the placenta plays crucial roles in the development and health of all offspring, but there is still little information on the human placenta.

Primary cultures, choriocarcinoma cell lines, and immortalized cell lines have traditionally been used in basic studies of the human placenta. However, it is been difficult to maintain primary trophoblast cells in culture, and various molecular mechanisms may be disrupted in choriocarcinoma and immortalized cell lines. Alternatively, mice have also been used to study the placenta because of their genetic uniformity and the ease of genome manipulation. Moreover, the availability of mouse trophoblast stem (TS) cells has greatly advanced research on the molecular mechanisms of trophoblast proliferation and differentiation.^5^ However, placental anatomy and trophoblast cell types are significantly different between mice and humans.^6^ Therefore, the establishment of human TS cells has long been awaited, but not achieved for about 20 years after the establishment of mouse TS cells. Recently, however, we succeeded in the establishment of human TS cells. Moreover, due to advances in bioengineering technologies and three‐dimensional culture systems, artificial embryos and trophoblast and endometrium organoids have become available.

In this review, we discuss the differences in the anatomy, transcription factor networks, and epigenetic properties between mice and humans during placental development and then describe the molecular pathology of perinatal complications. Finally, we introduce several recent technical breakthroughs in the field of human placental research.

## 2 PLACENTATION DURING EARLY PREGNANCY

Studies on the human placenta have been conducted using samples obtained from artificial abortion or preterm delivery. Animal models, such as the mouse, are also useful in elucidating the molecular mechanisms of placentation. However, it should be noted that although preimplantation development is similar between humans and mice, there are substantial differences in placental structure and trophoblast subtypes after implantation.

### 2.1 Placental development before and during implantation

**FIGURE 1:**
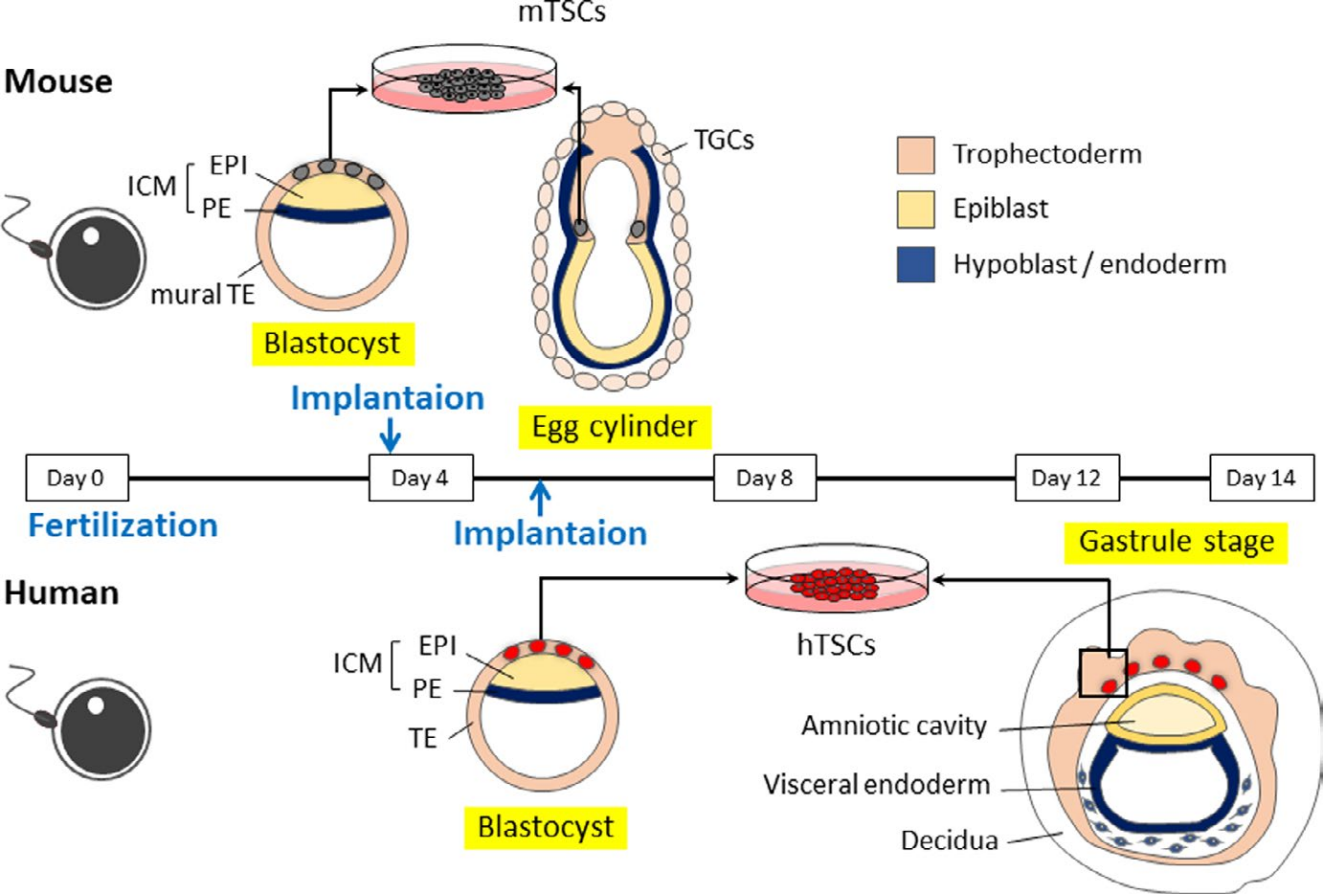
Comparison of peri‐implantation embryos in human and mouse. Epiblast and trophoblast directly interact in mice, but not in human. In mice, FGF4 from the epiblast is required for trophoblast proliferation. In humans, epiblasts and trophoblasts are separated by mesenchymal cells and cannot interact directly. Therefore, human trophoblast proliferation is likely to be epiblast (FGF4) EVT independent (maintenance of human TS cells requires EGF, not FGF). EPI: epiblast; PE: primitive endoderm; TE: trophectoderm; TSCs: trophoblast stem cells; TGCs: trophoblast giant cells

The mature oocyte and sperm fertilize to form a totipotent zygote. After several cell divisions, a zygote gives rise to a blastocyst that is composed of inner cell mass (ICM) and surrounding trophectoderm (TE) (Figure 1). TE is classified into the polar and mural TE: The former is adjacent to ICM, and the latter is not in contact with ICM. Whereas the polar TE proliferates rapidly, the mural TE stops cell division and differentiates into primary trophoblast giant cells (TGCs) in mice. TGCs are multinucleated cells that replicate their genomic DNA without cell or nuclear division. ICM differentiates into epiblast and primitive endoderm, which are destined to form embryonic and yolk sac tissues, respectively. Mouse blastocysts implant in utero at around embryonic day 4.5 (E4.5). During implantation, endometrial stromal cells undergo a special process called decidualization. This response is driven by the sex steroid hormones, estrogen, and progesterone, in both mice and humans.^7^ Decidualization begins after implantation in mice, whereas it is induced during the mid‐secretory to late secretory phase of the menstrual cycle irrespective of implantation in humans.^7^, ^8^ The interaction between trophoblasts and maternal decidua is particularly important for placental development.^9^ Impaired decidualization, such as endometriosis, has been reported to increase the risk of infertility and perinatal complications.^7^, ^10^

### 2.2 Placental development after implantation in mice

**FIGURE 2:**
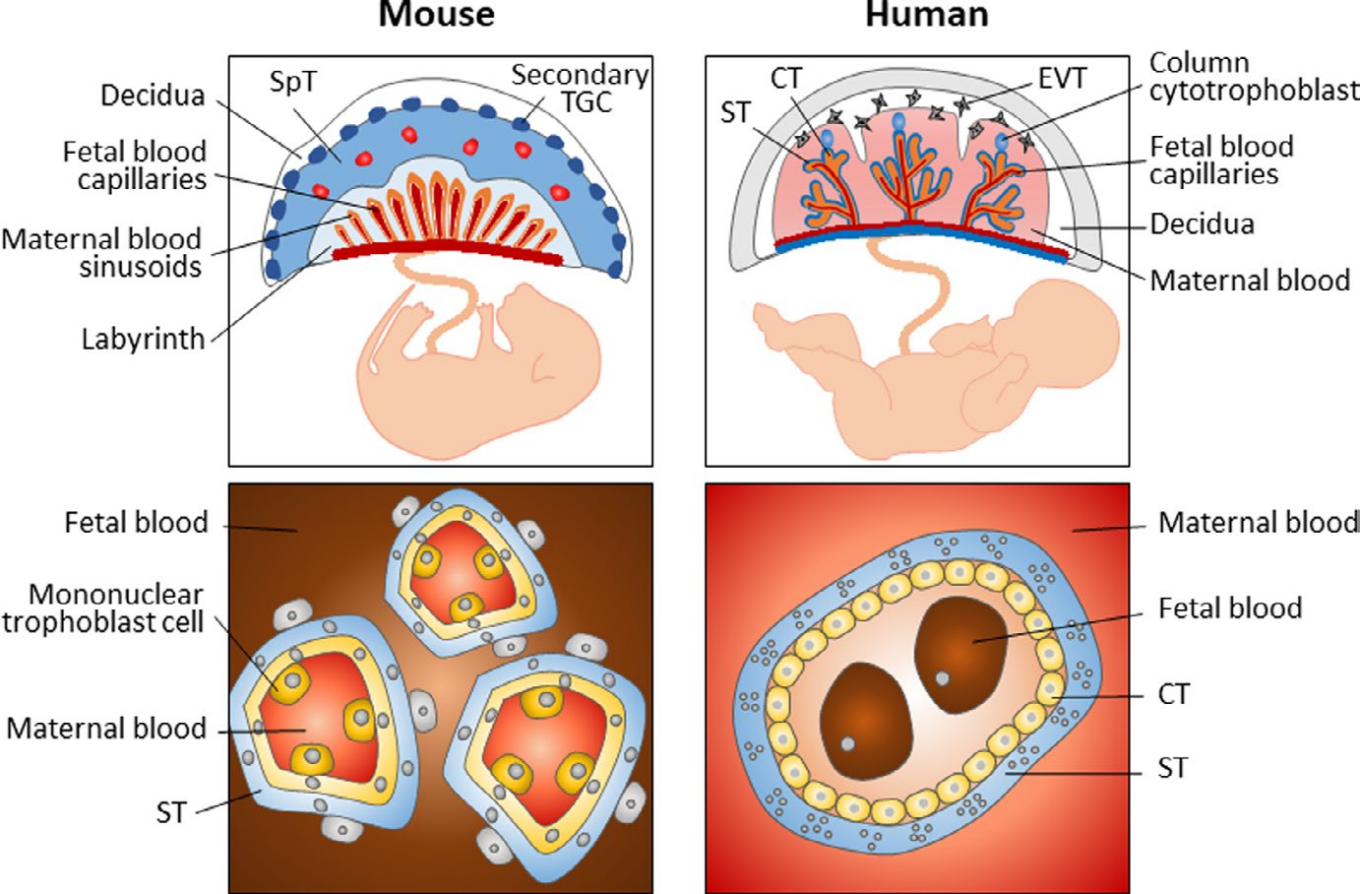
Structure of human and mouse mature placenta. Structure of the mouse placenta. The inset details the fetal‐maternal interface in the labyrinth. Structure of the human placenta. The inset image shows a cross section of a chorionic villus; trophoblast‐derived structures (blue) and mesoderm‐derived tissues (orange). The inset images illustrate the number and type of cell layers between the maternal and fetal blood. CT, cytotrophoblast; EVT, extravillous cytotrophoblast; SpT, spongiotrophoblast; ST, syncytiotrophoblast; TGC, trophoblast giant cell

In mice, a longitudinally elongated structure, known as the egg cylinder, is formed at about E6.0 (Figure 1). The extraembryonic ectoderm and the ectoplacental cone protrude into the maternal decidua. At the periphery of the ectoplacental cone, secondary TGCs differentiate. FGF4 provided by ICM and epiblast is required for the proliferation of polar TE and extraembryonic ectoderm.^5^ At E7.5, the ectoplacental cavity is formed. The base of the ectoplacental space is called the chorion. In the extraembryonic cavity, the extraembryonic mesoderm‐derived allantois extends from the fetal tail toward the chorion. At E9.0, the allantois fuses to the chorion and fetal blood vessels develop. At this stage, the chorionic ectoderm fuses with the roof of the ectoplacental space, and the ectoplacental space disappears. Failure of this process is the most frequent cause of mid‐pregnancy embryo lethality in mouse mutants.^11^, ^12^ By E10.0, the basic structure of the so‐called chorioallantoic placenta completes (Figure 2). The outermost layer (maternal side) of the placenta consists of secondary TGCs and serves to anchor the placenta to the uterus. A spongiotrophoblast layer is formed inside TGCs. After approximately E13.5, the spongiotrophoblast layer contains glycogen cells. The fetal side of the placenta, the labyrinthine layer, is invaded by fetal blood vessels from the allantois to form a capillary network.

### 2.3 Placental development after implantation in humans

In humans, implantation occurs at E6.0‐8.0. Trophoblasts adhere to endometrial epithelial cells and penetrate decidualized endometrial stroma. In mice, this invasion is triggered by TGCs, but in humans, primitive syncytiotrophoblast cells appear around undifferentiated cytotrophoblast (CT) cells and invade the decidua.^13^, ^14^ Primitive syncytiotrophoblast cells secrete enzymes that digest the decidua and enlarge the space for the embryo. In contrast to the mouse epiblast that acquires a columnar morphology, the human epiblast forms a disk‐like structure. Although the gross morphology is different, both mouse and human epiblasts form pseudostratified columnar epithelium. In the human embryo, epiblast cells adjacent to the trophoblast form squamous epithelium known as amnion and primitive endoderm‐derived yolk sac is formed. By E14.0, primitive streaks appear, gastrulation starts, and primordial germ cells (PGCs) are specified.

The basic structure of the human placenta is formed by about week 4 of gestation, and the maternal blood supply to the placenta is established by about 10 to 12 weeks (Figure 2).^15^ The mature placenta consists of three types of trophoblast cells: cytotrophoblast (CT), extravillous cytotrophoblast (EVT), and syncytiotrophoblast (ST). CT cells are highly proliferative epithelial cells that can differentiate into EVT and ST cells. EVT cells differentiate from CT cells at the tips of villi, invade the endometrium, and remodel the spiral arteries to control the maternal blood flow. These cells infiltrate to one‐third of the thickness of the uterine wall. ST cells are multinucleated cells formed by the fusion of CT cells and mediate nutrient and gas exchange between the fetal and maternal blood circulation. ST cells are also involved in hormone production. In addition to these trophoblasts, placental villi also contain ICM‐derived fibroblasts, vascular endothelial cells, and macrophages called Hofbauer cells. These cells are collectively called stromal cells.

Although the mouse and human placentas are morphologically different, they are both classified as the hemochorial placenta.^16^, ^17^ In humans, the major sites of maternal nutrient and gas uptake are lined by a single layer of ST cells. In mice, there are two layers of syncytium. The feto‐maternal interface is highly variable among mammalian species and believed to be optimized by species.^18^ At this interface, placental development proceeds through complex and sophisticated interactions between the trophoblast and the endometrium. Indeed, the maternal endometrium is known to play important roles in placental development. A study using human placental explant culture showed decidual stroma cell (DSC)‐derived Neuregulin‐1 (NRG‐1) promotes EVT differentiation.^19^ Moreover, DSCs secretes a variety of growth factors and cytokines. They alter the expression of MMPs and integrins in trophoblast, which promotes trophoblast invasion.^20^ At the same time, DSCs also secretes inhibitors of MMPs which suppress excessive trophoblast invasion.^21^ Thus, the trophoblast differentiation and invasion into maternal tissues are thought to be exquisitely controlled in part by the paracrine factors released from DSCs.^22^ The endometrial epithelium also plays an important role for placentation. As described above, the maternal arterial circulation to the placenta is not fully established until 10‐12 weeks of pregnancy. Endometrial glands are thought to be necessary to provide nutrients for the conceptus before the maternal blood supply. Indeed, the intervillous space before the entry of the maternal spiral artery is filled with secretions from the endometrium.^23^ Endometrial glands secrete amino acids, lipids, proteins, and sugars that are nutrients of the conceptus.^24^ Endometrial glands also supply growth factors such as VEGF, LIF, and EGF.^25^ Studies using human villus explants showed that EGF stimulates CT cell proliferation at 4‐5 weeks of gestation and the secretion of hCG and human placental lactogen (hPL) from ST cells at 6‐10 weeks of gestation.^26^ Thus, secretion from both of stromal cells and epithelial cells is important at each stage of trophoblast growth and differentiation.

## 3 TRANSCRIPTION FACTOR NETWORKS REGULATING TROPHOBLAST DEVELOPMENT

In mouse preimplantation embryos, Oct4 (Pou5f1) and Cdx2 antagonize each other and play an important role in the segregation of the embryonic and extraembryonic lineages.^27^, ^28^ Oct4 protein is expressed in all cells of the morula but gradually restricted to ICM. Cdx2 is expressed in the outer cells of the morula and completely restricted to TE at the blastocyst stage.^29^ The Hippo signal transcriptional coactivator Yap preferentially induces preferential differentiation into TE. Yap translocates to the nucleus of the cells on the outer cells of the morula and activates the expression of the transcription factors Tead4 and Cdx2 in mice.^30^, ^31^ CDX2 and OCT4 exhibit similar expression patterns in human blastocysts,^32^ but YAP is localized to the nucleus in both ICM and TE at the late blastocyst stage. TEAD4 expression, on the other hand, seems to show well‐conserved expression patterns between mice and humans. The role of Hippo signaling during human preimplantation development has not been well studied.^33^ Whereas Cdx2 expression persists in the extraembryonic ectoderm after implantation in mice, it is controversial whether CDX2 is expressed in trophoblast lineages after implantation in humans.^34^, ^35^, ^36^

**FIGURE 3:**
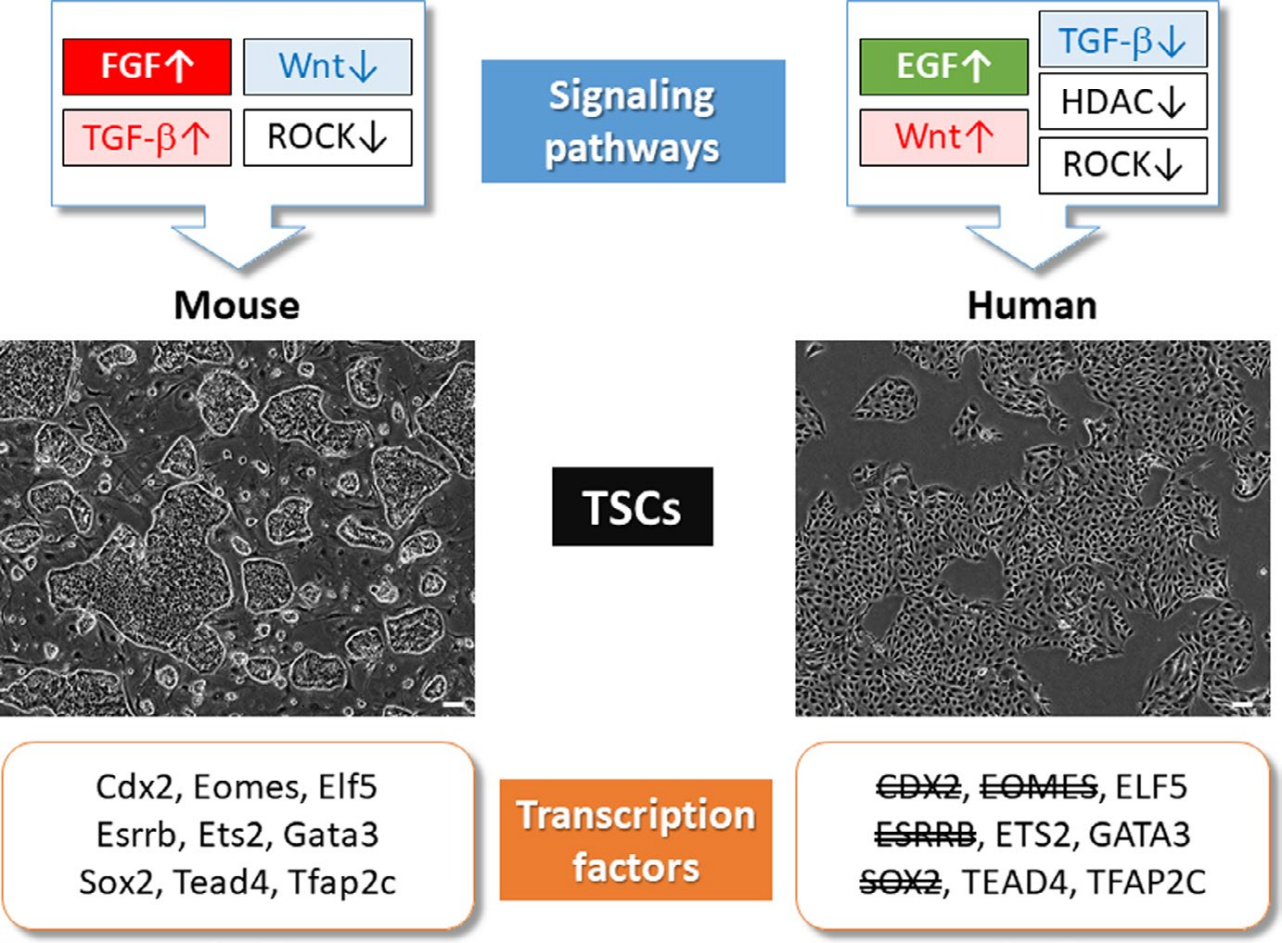
Comparison of signaling pathway and transcription factors between human and mouse TSCs. Wnt and TGF‐β signals are inversely involved in mTSCs and hTSCs. Some of the transcription factors required to maintain mTSCs are not expressed in hTSCs. In mTSCs, Esrrb and Sox2 are known to be activated downstream of FGF signals. ESRRB and SOX2 are not expressed in hTSCs, which is consistent with the FGF‐independent nature of hTSCs. The specific transcription factors for hTSCs are currently unknown

During mouse placental development, Tead4 and Cdx2 act in concert with multiple transcription factors, including Eomes, Elf5, Ets2, Esrrb, Gata3, Sox2, and Tfap2c.^37^, ^38^ Dysfunction of these genes leads to abnormal placentation and embryonic lethality shortly after implantation.^39^, ^40^, ^41^, ^42^, ^43^, ^44^, ^45^, ^46^, ^47^, ^48^, ^49^, ^50^ Mouse TS cells have been used to analyze the interactions and regulation of these transcription factors. For example, Esrrb and Sox2 are found to be targets of the fibroblast growth factor (FGF) signaling mediated by Fgfr2c. Removal of FGF or addition of FGF signaling inhibitors rapidly reduces TS cell‐specific gene expression. Interestingly, the expression of ESRRB and SOX2 is negligible in TE and CT cells in humans. This observation is consistent with the absence of FGFR2C in human TE and CT cells.^44^, ^45^, ^51^ Therefore, ESRRB and SOX2 might be dispensable for human trophoblast development. Additionally, human TE and CT cells do not express EOMES.^32^, ^35^, ^52^ In contrast, GATA3, TFAP2C, ELF5, and ETS2 are expressed in TE and/or CT cells in humans.^32^, ^35^, ^36^, ^53^, ^54^ These differences and similarities of the expression patterns of transcription factors in human and mouse trophoblast cells are summarized in Figure 3.

Most of the transcription factors that regulate trophoblast development do not show trophoblast‐specific expression patterns. In mice, all transcription factors described above are also expressed in non‐trophoblast cells. It is been recognized that transcription factors can change their partners in a cell type‐dependent manner. For example, Sox2 and Esrrb are essential for both mouse embryonic stem (ES) and TS cells. Sox2 forms a complex with Tfap2c in TS cells,^44^ but Sox2 interacts with Oct4 in ES cells. Esrrb interacts with histone demethylase Kdm1a and RNA polymerase II‐associated integrator complexes in TS cells but not in ES cells.^45^ Another important aspect is the relative ratio of interacting transcription factors. Additionally, the EOMES‐ELF5 protein complex acts to inhibit differentiation of mouse TS cells,^55^ and the TFAP2C‐ELF5 protein promotes differentiation.^56^ Collectively, the transcription factor networks regulating mouse TS cells have gradually been understood. However, little is known in humans.

## 4 EPIGENETIC REGULATION IN THE PLACENTA

Epigenetics is important not only for cell fate determination but also for stabilization of lineage‐specific gene expression patterns. Genetic information must be properly regulated by epigenetic modifications.^57^ Epigenetic modifications such as DNA methylation and histone modifications play crucial roles in placental development.

### 4.1 DNA methylation reprogramming after fertilization

**FIGURE 4:**
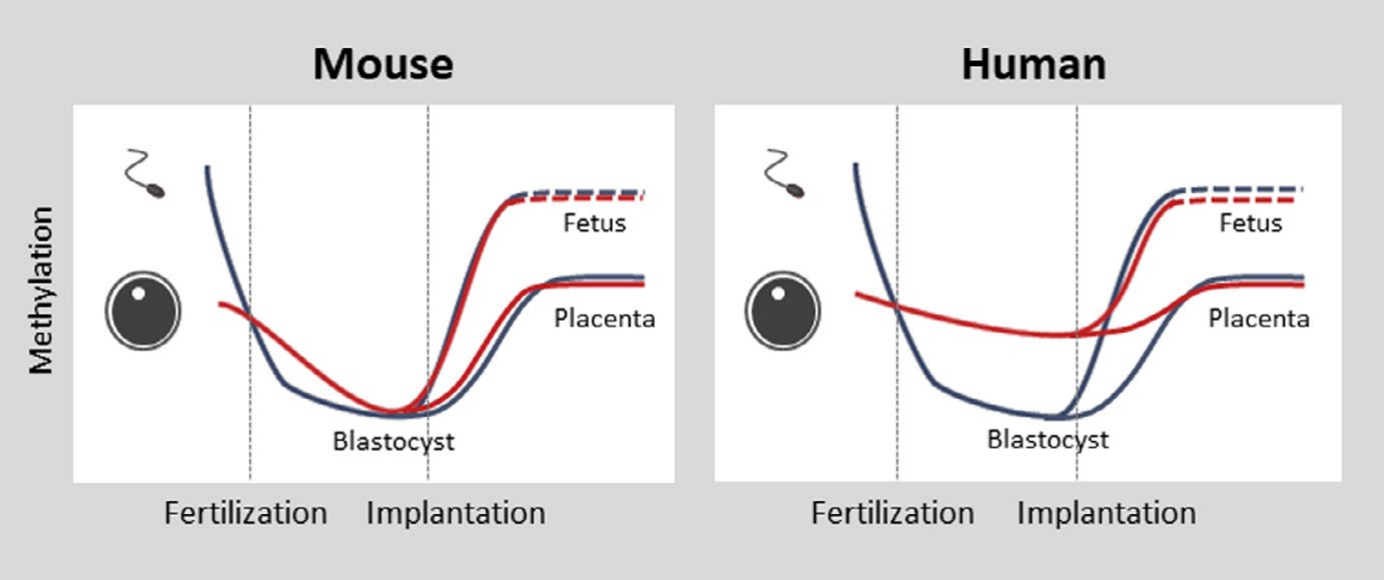
Dynamic DNA methylation change of early embryo in human and mouse. The maternal genome was demethylated to a much lesser extent in human blastocysts than in mouse blastocysts, which increase the number of imprinted DNA methylation

Among epigenetic modifications, methylation of cytosine residues in CpG dinucleotides has been well studied. DNA methylation plays important roles not only in cell lineage specification but also in the control of the genome imprinting and the X chromosome inactivation. DNA methylation goes through two dramatic reprogramming during mammalian development. One occurs during gametogenesis, and the other does after fertilization.^58^ These two waves of reprogramming are observed in both humans and mice. Global DNA demethylation during gametogenesis allows expression of genes required for meiosis and also resets the parent‐of‐origin‐dependent DNA methylation at the imprinted loci. Some retrotransposons are resistant to demethylation and mediate the inheritance of epigenetic information across generations.^59^ Global DNA demethylation also occurs after fertilization, but the imprinted loci are protected at this stage. In mice, the sperm‐derived genome is demethylated by the ten‐eleven translocation (Tet) 3 demethylase, which is known as active demethylation. On the other hand, the oocyte‐derived genome is protected from demethylation by Tet3. However, due to the weak activity of the maintenance DNA methyltransferase Dnmt1, methylated DNA is lost at each cell division, which is known as passive demethylation. DNA methylation patterns of sperm and oocytes are similar between humans and mice,^60^, ^61^, ^62^ and the sperm‐derived genome also go through active demethylation in human embryos. However, passive demethylation seems to be incomplete in humans, and the oocyte‐derived genome shows higher methylation levels in humans than in mice (Figure 4).

After implantation, DNA methylation is regained in both embryonic and extraembryonic cells, which is mediated by the de novo DNA methyltransferases (DNMT) 3A and DNMT3B^63^, ^64^, ^65^. However, whereas ~80% of CpG sites are methylated in epiblast cells, only 40‐50% of CpG sites are methylated in trophoblast cells. In both embryonic and extraembryonic tissues, the promoters are largely unmethylated and the gene body regions of actively transcribed genes are highly methylated. However, compared to epiblast cells, trophoblast cells have less DNA methylation at the gene body regions of weakly expressed genes and intergenic regions. The placenta is thought to have the lowest DNA methylation levels among tissues,^66^, ^67^, ^68^, ^69^ and the methylome of trophoblast cells are characterized by partial methylation domains (PMDs).^68^, ^70^, ^71^ These PMDs are the same in mice and humans, but their functional significance is unknown.

### 4.2 Regulation of placenta‐specific gene expression by DNA methylation

Many genes have been identified to show placenta‐specific expression,^72^ and some of them are regulated by DNA methylation. For example, the promoter of ELF5 is hypomethylated in trophoblast cells but hypermethylated in most embryonic lineage cells in both humans and mice.^36^ The promoters of INSL4 and DSCR4 show similar methylation patterns.^73^, ^74^ We also recently identified 55 promoters that exhibit placenta‐specific hypomethylation in humans.^52^

The DNA‐binding capacity of many transcription factors is influenced by DNA methylation. In vitro studies using synthetic methylated and unmethylated DNA‐binding domains have shown that the binding affinity of about 60% of human transcription factors is affected by DNA methylation.^75^, ^76^ Transcription factors that are important in trophoblasts, such as ETS2, ELF5, and TFAP2C, bind unmethylated DNA.

Long terminal repeat (LTR) sequences of endogenous retroviruses are associated with placental evolution in mammals. LTRs are rich in transcription factor binding motifs and exhibit unique functions in extraembryonic tissues.^77^, ^78^ DNA methylation is responsible for the stable silencing of foreign DNA.^79^, ^80^, ^81^ In mouse and human trophoblast cells, many LTRs are hypomethylated.^82^, ^83^ Recently, it has been reported that LTRs act to stimulate the secretion of corticotropin‐releasing hormone, a regulator of gestational age.^84^ It has also been reported that LTRs upstream of the nitric oxide synthase gene NOS3 drive placenta‐specific transcriptional isoforms in the human placenta. Moreover, syncytins, envelope genes derived from retroviruses, play an important role in both mouse and human ST cells.^85^, ^86^ Thus, retrotransposons and their DNA methylation patterns may contribute to the evolution of the placenta.

### 4.3 Development of trophoblast cells and histone modifications

Histone modifications are essential for trophoblast development in mice. Embryos deficient in EHMT2 (also known as G9A), which mediates H3K9 dimethylation, fail to fuse the chorioallantoic membranes, which is lethal in mid‐pregnancy.^87^ Polycomb group proteins responsible for H3K27me3 modification are essential for chorionic villus formation, and their defects result in abnormalities from early‐ to mid‐gestation.^88^, ^89^ H3K9 dimethylation, trimethylation (H3K9me 2/3), and H3K27me3 are inhibitory marks and often function in overlap with DNA methylation. Mouse ES cells deficient in H3K9 trimethylation‐mediated histone methyltransferase (SETDB1) express Cdx2 and differentiate into trophoblast cells.^90^, ^91^, ^92^ Lysine demethylase (KDM1A)‐deficient mouse ES cells exhibit similar characteristics. H3K27me3 and H3K9me2 are also involved in the regulation of imprinted genes in the mouse placenta.^93^, ^94^, ^95^ Despite these important findings in mice, the functional roles of histone modifications are poorly understood in human trophoblast cells.

### 4.4 Placenta‐specific genomic imprinting:

In mice, the development of both female‐developing embryos (embryos composed of the oocyte‐derived nuclei) and androgenetic embryos (embryos composed of sperm‐derived nuclei) is lethal. So, the development of these causes the death of the embryo. However, their phenotypes are very different. Parthenogenetic embryos rarely form the placenta, whereas androgenetic embryos show placental hyperplasia. Similarly, in humans, androgenesis is associated with complete hydatidiform mole, a disease characterized by trophoblast overgrowth. Thus, genomic imprinting is important for placental development.

Some imprinted genes show uniparental expression in a tissue‐specific manner, and many tissue‐specific imprinted genes have been found in mice, especially in the placenta and brain. We performed a comprehensive analysis of the imprinted genes in the mouse placenta,^96^ which revealed that many placenta‐specific imprinted genes reported by that time were due to contamination of maternal tissues, and only 12 genes actually underwent imprinting. We also demonstrated that uniparental expression of these placenta‐specific imprinted genes was disrupted in the placentas of somatic cell nuclear transfer (SCNT) mouse embryos, which are typically characterized by placental hypertrophy. These findings reveal the importance of placenta‐specific imprinted genes in the placental development.^97^, ^98^ However, human homologs of mouse placenta‐specific imprinted genes do not show uniparental expression in human placentas.^96^ There are about 110 human placenta‐specific imprinted genes, far more than the mouse imprinted genes.^64^ This is likely due to incomplete demethylation of the oocyte‐derived genome after fertilization in humans. In addition to protein‐coding genes and long non‐coding RNAs, some miRNAs are imprinted in the placenta. There are about 2000 miRNAs in the human and mouse genomes. MiRNAs bind to target mRNAs and inhibit translation. Some miRNAs form clusters, and the miRNA clusters on chromosomes 14 and 19 are regulated by genomic imprinting in the human placenta.^99^, ^100^, ^101^ The reason why humans and mice have acquired almost completely different sets of placenta‐specific imprinted genes is currently unknown, but it is possible that they contribute to differences in placental structure, gestational period, or litter size between these species.

### 4.5 Incomplete X chromosome inactivation in human placenta:

One of two X chromosomes is inactivated in female mammalian cells. In marsupials, both embryonic and extraembryonic tissues show imprinted X chromosome inactivation, in which the paternal X chromosome is selectively inactivated. In mice, inactivation of the X chromosome in embryonic tissues is random, whereas the paternal X chromosome is selectively inactivated in the placenta. It has been reported that X chromosome inactivation is skewed in the human placenta. The paternal X chromosome is preferentially inactivated in the human placenta but the degree varies among individuals.^64^, ^102^

## 5 EPIGENOMIC ABNORMALITIES AND PLACENTAL DISORDERS

Large‐scale knockout studies in mice indicate that about 25‐30% of all genes are essential for survival. However, with few exceptions, such analyses have focused on embryos and little attention has been paid to placentas.^103^ A recent study revealed that morphological abnormalities of the placenta are observed in approximately two‐thirds of embryonic lethal mutant mice.^12^ It is also known that placental abnormalities tend to be associated with abnormalities of the heart, brain, and vascular embryos. In humans, infants with congenital heart disease have also been reported to exhibit a significantly higher frequency of placental abnormalities than healthy controls.^104^, ^105^, ^106^

Abnormalities in genomic imprinting may be associated with the development of small for gestational age (SGA), intrauterine growth restriction (IUGR), and HDP in humans. For example, in the placenta of IUGR, there is a trend toward increased expression of the paternally imprinted gene PHLDA2 and decreased expression of the maternally imprinted genes MEST and PLAGL1. Decreased expression of paternally imprinted genes MEG3 and GNAS has also been reported.^121^

**TABLE 1:** Previous *in vitro* models for human trophoblast cell research

Name	Origin	Established methods	Marker	Section references
Trophoblast cell line	First‐trimester placenta	Explants	CK, hCG	107
Long‐term cytotrophoblast culture	First‐trimester placenta	Explants	CK, hCG, Trop‐1, Trop‐2	108
HTR‐8/SVneo	Early placenta	SV40 large T antigen （immortalization）	CK, hCG	109
HT	Full‐term placenta	Primary culture (transformed trophoblasts)	CK, hCG, Placental alkaline phosphatase, Trop‐2	110
TCL‐1	Chorionic membrane of placenta	SV40 large T antigen（immortalization）	hCG	111
NPC	First‐trimester placenta	EGF, Insulin, Dexamethasone	CK18, GnRH, Neuropeptide Y, Neurotensin, Leucine‐enkephalin, Dopamine, 5‐HT, Progesteron, hCG	112
IST‐1	First‐trimester placenta	Retrovirus encoding HPV16 E6 and E7 proteins（immortalization）	CK7, CK18, hPL, Mel‐CAM (CD146)	113
BMP4‐hESC	ES cell	BMP (FGF2, MEF‐CM)	TFAP2, MSX2, SSI3, GATA2, GATA3, HEY1, CG‐α, CG‐β	114
CTBS	ES cell	Embyoid body formation, FGF4, heparin, MEF‐CM	CDX2, HLA‐G, CD9,CK7	115
HPT‐8, HPT‐8‐HBV	First‐trimester placenta	Primary culure, singel cell cloning, HBV （immortalization）	CK7, CK18, Vimentin, CD9, EGFR, SDF1, Prolactin, E2, Progesterone, hCG, HLA‐G	116
TBPCs	Chorion membrane of first‐trimester placenta	FGF2, SB431542 (TGF‐β inhibitor), gelatin substrates	OCT4, ZO‐1, GATA4, Nestin, CK7	117
iTP	Human fetal fibroblast (IMR90)	Lentivirus（CDX2, ELF5, C‐MYC, KLF4, EOMES）	CDX2, EOMES, ELF5, CK7, GATA3, TEAD4	118
BAP‐hESC	ES cell	BMP4, A83‐01 (ALK4/5/7 inhibitor), PD173074 (FGF2‐signaling inhibitor), MEF‐CM	CK, T, HLA‐G	119
TSCs from UCSFB	ES cell lines from blastomeres	Embryoid body formation, FGF2, SB431542	CDX2, TEAD4, GATA3, ELF5, GDF15, β‐catenin	120

HDP is also thought to be associated with epigenetic abnormalities. The expression of several miRNAs is reported to be dysregulated in preeclamptic placentas compared to normal placentas.^122^, ^123^ The human placenta‐specific imprinted gene CUL7 is also reported to be hypomethylated and shows increased expression in human fetal growth restriction placentas.^124^ Deficiency of CUL7 also causes fetal growth restriction, severe post‐natal growth restriction, and 3M syndrome type I, a congenital anomaly syndrome characterized by characteristic facies.^125^ Furthermore, it has been reported that Cul7‐deficient mice cause vascular abnormalities in the decidua and exhibit phenotypes such as impaired placental development and fetal growth restriction.^124^ CYP2J2, which is a placenta‐specific imprinted gene^64^ and encodes one of the cytochrome P 450 enzymes known as drug‐metabolizing enzymes,^126^ is reported to be highly expressed in HDP patients.^126^ In addition, the metabolite EET of CYP2J2 is also increased in a rat model of HDP. These results support the hypothesis that aberrant expression of imprinted genes may be associated with IUGR and HDP.

A complete hydatidiform mole (CHM) is caused by androgenesis and is characterized by abnormal proliferation of placental trophoblasts. CHM is also associated with a high rate of secondary tumors and cancers, making this disease one of the most important pregnancy complications for clinical diagnosis and management. Abnormalities in the expression of imprinted genes have been thought to play a major role in the pathogenesis of CHM, but it is not clear which imprinted genes are involved in the pathogenesis. Recently, we established cell lines from CHM samples and demonstrated that silencing of the imprinted gene P57KIP2 confers resistance to cell cycle arrest by contact inhibition in these cell lines.^127^

## 6 DEVELOPMENT OF USEFUL TOOLS TO STUDY HUMAN PLACENTAL DEVELOPMENT

**FIGURE 5:**
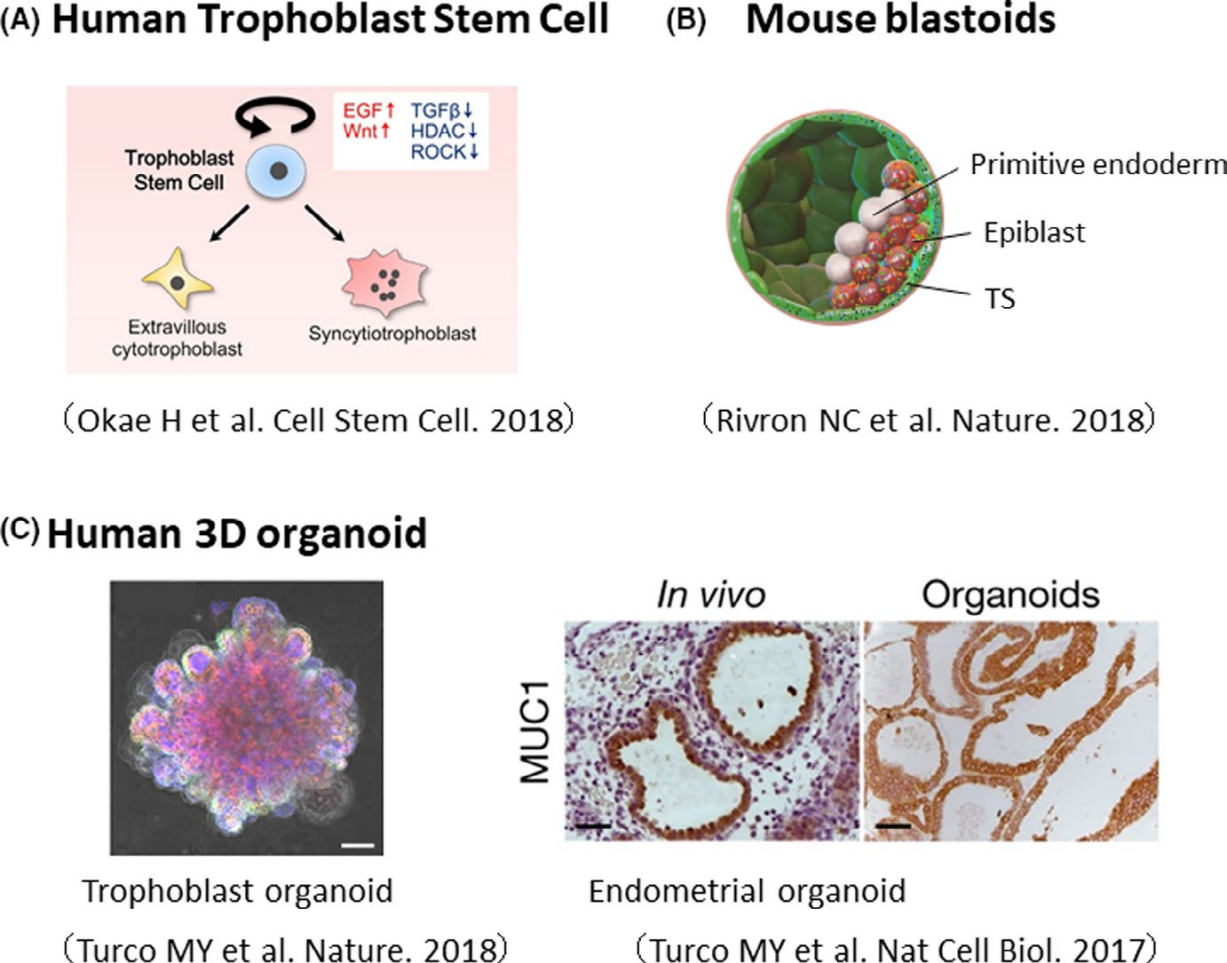
Development of useful tools to study human placental development

The establishment of mouse TS cells was first reported in 1998.^5^ It was only recently that human TS cells were established.^52^ Furthermore, in recent years, various useful tools have been developed to study placental development, such as artificial mouse embryos and human trophoblast and endometrial organoids (Figure 5).

### 6.1 Human trophoblast stem (TS) cells:

In 1998, mouse TS cells were first derived from blastocysts or extraembryonic ectoderm of post‐implantation embryos using FGF4, heparin, and mouse embryonic fibroblasts (MEF).^5^ However, the culture conditions of mouse TS cells cannot be applied to human TS cells, probably due to the differences in the molecular mechanisms regulating trophoblast proliferation and differentiation. For example, FGFR2c, a receptor for FGF4, is expressed in mouse blastocysts but not in human blastocysts.^51^ Although various models have been generated and used to study human trophoblast cells (Table 1), the establishment of human TS cells has not been achieved for a long time.

We recently succeeded in establishing human TS cells from blastocysts and CT cells of first‐trimester placental villi. Human TS cells can be maintained in an undifferentiated state for a long period (80 passages or more) and meet following four criteria for trophoblast cells.^52^, ^53^ (1) expression of trophoblast markers such as GATA3 and TFAP2C; (2) decreased expression of HLA class I molecules; (3) hypomethylation of the *ELF5* gene promoter; and (4) expression of the placenta‐specific miRNA cluster C19MC. We found that activation of Wnt and EGF signaling and inhibition of TGF‐β signaling, HDAC, and ROCK are important for human TS cell derivation. When treated with the adenylate cyclase activator forskolin, human TS cells fuse to differentiate into multinucleated ST cells. Human TS cells can also differentiate into spindle‐shaped extravillous cytotrophoblast (EVT) cells when they are treated with neuregulin (NRG1) and a TGF‐β inhibitor. The methylation patterns of human TS cells are highly correlated with those of primary trophoblast cells. Furthermore, when human TS cells were implanted subcutaneously in immunocompromised mice, they infiltrated the dermis and subcutaneous tissue of the mice and differentiated into EVT‐ and ST‐like cells. Interestingly, some of the ST‐like cells were found to be vacuolated and have an influx of mouse blood flow. This structure closely resembles the primordial syncytial cells, which are specialized cells produced when human blastocysts implant in the uterus. In summary, human TS cells retain unique characteristics of trophoblast cells and therefore are very useful to analyze human placental development and function.

### 6.2 Artificial embryos generated using TS and ES cells:

TS cells have also been used for modeling of early embryos. Mouse TS cells, when combined with ES cells, self‐organize to form post‐implantation embryo‐like structures that mimic early embryo development.^128^ Blastocyst‐like structures were also generated using mouse ES and TS cells. These “blastoids” have the potential to implant into the mouse uterus and induce decidualization. Although ethical issues must be addressed, the development of human artificial embryos using TS and ES cells will open up great potential for the study of human embryogenesis.

### 6.3 3D trophoblast organoids

Soon after human TS cell establishment was reported, three‐dimensional trophoblast organoids have been generated using CT cells purified from first‐trimester placental tissue.^129^, ^130^ These organoids contain CT‐ and ST‐like cells and can be maintained for a long time. They also give rise to EVT‐like cells as human TS cells do. Such trophoblast organoids are useful for studying placental development and function under more physiological conditions.

### 6.4 New models and data resources to study the feto‐maternal interface

The generation of organoid models has also been reported for the maternal endometrium. Three‐dimensional culture of endometrial glands leads to self‐organized cyst‐like structures, which respond to sex hormones such as estrogen and progesterone. Endometrial organoids can also replicate the phenotype of endometriosis and endometrial cancer.^131^ Endometrial receptivity is essential for successful implantation and pregnancy. Indeed, two‐thirds of implantation failure is caused by endometrial receptivity.^132^ The combination of organoid technologies that mimic the development of embryos, trophoblast cells, and endometrium has the potential to recapitulate implantation and placentation more precisely than the conventional trophoblast‐endometrium coculture assay.^133^, ^134^ This would expand our understanding of the human early developmental process, which has been a “black box” due to ethical constraints.

Recently, single‐cell RNA‐seq analysis has provided a comprehensive picture of the cell populations present in the human placenta and decidua. This approach also enables the identification of previously unknown cell populations. Moreover, the analysis of ligand‐receptor interactions based on the expression data makes it possible to estimate the dynamic interactions between fetal and maternal cells at the feto‐maternal interface.^135^, ^136^

## 7 CONCLUSIONS AND FUTURE PERSPECTIVES

Maternal nutrition, physical activity, and psychological stress during pregnancy can affect not only fetal and placental growth but also the risk of cancer and lifestyle‐related diseases such as cardiovascular disease and diabetes in adulthood. It is known as the “Developmental Origins of Health and Disease (DOHaD)” theory.^137^, ^138^ The placenta is sensitive to environmental changes in utero and may be involved in the lifelong health of humans. Studies of the placenta have made significant progress, led by studies on mouse models. However, as we have seen, the placental structure and development differ greatly between humans and mice. The recent establishment of human TS cells and trophoblast and endometrial organoids enhances our understanding of the mechanisms underlying human placental development. Moreover, further advancement of these techniques will lead to a better understanding of embryogenesis and implantation and the treatment of diseases such as infertility and pregnancy‐induced hypertension.

## Disclosures

Our derivation of human TS cells was approved by the Institutional Review Board (IRB) approval at Tohoku university (2014‐1‐879).

*Conflict of interest*: Authors have no conflict of interest to be declared.
